# A Monster

**Published:** 1866-12

**Authors:** 


### A MONSTER.

The following short account of a singular case of labor, and the birth of an extraordinary monster, was sent us by Dr. J. K. Hamilton, of Stone Mountain, Ga.

We very much regret not being able to obtain the much desired examination of the body, in order to ascertain the direction of the cord from the point of attachment, and the exact nature of the other peculiarities connected with the formation of this child.

---

*Editors Medical Journal:*

I was called, on the third of April last, to see Mrs. N., who was in labor with her first child, eight months *enciente.*

I made a vaginal examination, and discovered a case of placenta prævia. The pains were regular and persistent, with slight protrusion of placenta during paroxysms. Considerable hemorrhage ensued, which was partially controled by the tampon, cold applications and rest. The labor lasted about two hours: the afterbirth emerged first, and was followed almost immediately by the expulsion of the child.

The most remakable feature of the case was: The umbilical cord was attached to the crown of the head, leading directly from the placenta, seeming to permeate the brain, or more probably the inner surface of the scalp. The neck was unusually large, caused probably by an undue supply of vascularity and nervous influence, with a consequent developement of tissues surrounding them.

There was a cleft in the upper lip, constituting simple hare-lip. The abdomen contained a fissure extending from the epigastrium to near the symphesis pubis; hence the child was nearly disemboweled, with apparent obliteration of the umbilicus. The liver and intestines were well developed, and although it exhibited evidences of recent vitality, it came still-born, owing, doubtless, to the anomalous attachment of the placenta and cord. The father of the child, during the late war, lost his left forearm, in Virginia, it being amputated about six inches below the elbow: the child, also, on same side, had its forearm off—the stump bearing a great similarity to the arm of the father.

The assimilation process in this instance, as respects growth and development, was normal, the trunk and limbs being properly proportioned.

I do not propose, in this short report, to attempt to explain the causes which may give rise to these preternatural perversions of the laws of the animal economy. In one respect it may have ensued from some accidental change of position experienced by the fœtus at some period of its uterine existence; and, in another, it may have originated from the influence of the maternal imagination on the fœtus in utero, or attributable to a primitive defect in the germ. Whilst we know and appreciate the opinions of learned physiologists, in regard to the causes of monstrosity, further, deponent saith not.

J. K. Hamilton, M. D.

